# Racial Differences in Age at Diagnosis and Renal Manifestations of Antineutrophil Cytoplasmic Antibody (ANCA)-Associated Vasculitis: Insights From a Multi-institutional Electronic Health Record Retrospective Cohort

**DOI:** 10.7759/cureus.111279

**Published:** 2026-06-22

**Authors:** Muhammad Siddiqi, Siddharth Nara, Amber Paulus, Jason M Kidd

**Affiliations:** 1 Division of Nephrology, Virginia Commonwealth University School of Medicine, Richmond, USA

**Keywords:** acute kidney failure, anca-associated vasculitis, health disparity, inequities, kidney failure

## Abstract

Background: Antineutrophil cytoplasmic antibody (ANCA)-associated vasculitis (AAV) is a rare autoimmune disease in which renal involvement is a major determinant of morbidity and mortality. Epidemiologic data suggest lower reported prevalence of AAV among Black populations, but whether differences exist in disease presentation and renal severity at diagnosis remains poorly characterized.

Methods: We conducted a retrospective cohort study using a large, multi-institutional electronic health record network. Adults diagnosed with AAV between 2015 and 2025 were identified using International Classification of Diseases, Tenth Revision (ICD-10) codes. Demographic characteristics, renal manifestations at presentation, dialysis dependence, and ANCA serologic testing were extracted. Primary analyses compared Black and White patients using descriptive statistics, Welch t-tests, Fisher’s exact tests, and odds ratios (ORs) with 95% confidence intervals (CIs).

Results: Among 789 adults with AAV, 84.8% were White and 8.2% were Black. Black patients were diagnosed at a younger mean age than White patients (55 ± 15 vs 64 ± 16 years; p<0.001). Dialysis dependence was more frequent among Black patients (21.5% vs 13.3%; OR: 1.79, CI: 0.88-3.45), as was hematuria (38.5% vs 28.1%; OR: 1.59, CI: 0.9-2.78). Proteinuria occurred at similar rates across groups. ANCA serologic testing was incompletely captured.

Conclusions: In this multi-institutional cohort, Black patients with AAV presented at a younger age and tended to be more likely to require dialysis and have hematuria. These findings suggest that underrepresentation in rare disease cohorts may coexist with substantial disease burden at presentation, underscoring the importance of examining severity alongside prevalence in population-level studies.

## Introduction

Antineutrophil cytoplasmic antibody (ANCA)-associated vasculitis (AAV) is a rare, systemic autoimmune disease characterized by necrotizing inflammation of small- to medium-sized blood vessels, often with renal involvement, frequently manifesting as rapidly progressive glomerulonephritis [1-4]. AAV encompasses a heterogeneous spectrum of clinical phenotypes, including granulomatosis with polyangiitis, microscopic polyangiitis, and eosinophilic granulomatosis with polyangiitis, which share overlapping pathophysiologic mechanisms driven by ANCA-mediated neutrophil activation and endothelial injury [2-6].

Kidney involvement is a defining and prognostically important feature of AAV and strongly influences both short- and long-term outcomes [3,4,7]. Severity of renal disease at presentation, including reduced kidney function, active urinary sediment, and dialysis dependence, is among the strongest predictors of mortality and progression to end-stage kidney disease, even in the modern treatment era [7-10]. Although advances in immunosuppressive therapy have substantially improved survival, patients with AAV continue to experience excess morbidity and mortality compared with the general population [8,10-13].

Epidemiologic studies suggest that AAV is more commonly reported in White populations, with lower observed prevalence among racial and ethnic minority groups; however, the underlying drivers of these differences remain poorly understood [1,3,4]. Existing literature is limited by small sample sizes, geographic variation, and underrepresentation of diverse populations in observational cohorts and clinical trials, resulting in an incomplete understanding of whether differences exist in disease recognition, timing of diagnosis, or severity at presentation across racial groups [3,14].

Current clinical practice guidelines emphasize that early recognition of AAV and prompt initiation of immunosuppressive therapy are critical for preserving kidney function and improving survival, particularly given the potential for irreversible renal injury [12,15,16]. Delayed or missed diagnosis remains common, especially among patients presenting with non-specific symptoms or renal-predominant disease, which may lack overt symptoms early in the disease course [5,11]. Decreased recognition and consideration of these disease processes in historically underrepresented populations may contribute to inequities in outcomes.

The objective of this study was to explore differences in demographic distribution, renal manifestations, and dialysis dependence among adults diagnosed with AAV using a large, multi-institutional electronic health record (EHR) cohort.

## Materials and methods

Study design and data source

We conducted a retrospective cohort study using data from Epic Cosmos, a large, federated EHR database that aggregates longitudinal, de-identified inpatient and outpatient data from participating Epic health systems across the United States. Epic Cosmos integrates encounters across care settings and institutions, capturing diagnoses, laboratory results, procedures, medications, and selected social and clinical variables as patients move between health systems. The study population included adults (≥18 years) with a documented diagnosis of AAV between January 1, 2015 and April 10, 2025.

Study population

Patients were identified using International Classification of Diseases, Tenth Revision (ICD-10) diagnostic codes for ANCA-associated vasculitis (I77.82).

Variables

Extracted variables included demographic characteristics (age at diagnosis, sex, and race), renal manifestations at presentation (hematuria, proteinuria, and abnormal serum creatinine), dialysis dependence at presentation, ANCA serology (proteinase 3 (PR3) and myeloperoxidase (MPO), identified using Logical Observation Identifiers Names and Codes), and renal biopsy status (Current Procedural Terminology code 50200). Race was categorized based on EHR documentation. Primary analyses focused on comparisons between Black and White patients. Medications used in the management of AAV were explored but incompletely captured and thus cannot be evaluated. 

Statistical analysis

Descriptive statistics were used to summarize demographic and clinical characteristics. Continuous variables are reported as mean ± standard deviation, and categorical variables are reported as counts and percentages. Differences in age at diagnosis between racial groups were assessed using a two-sample Welch t-test based on summary statistics, given unequal sample sizes and variances. Comparisons of categorical variables were performed using Fisher’s exact test due to small cell counts. Odds ratios (ORs) with 95% confidence intervals (CIs) were calculated from contingency tables to quantify the magnitude of associations. All analyses were restricted to comparisons between Black and White patients and were conducted using R statistical software (R Foundation for Statistical Computing, Vienna, Austria).

## Results

The study cohort comprised 789 adults of whom 669 (84.8%) were White and 65 (8.2%) were Black or African American (Table 1). Women accounted for 57.7% of the total population. Among White patients, 56.4% were female, compared with 66.2% among Black patients.

**Table 1:** Demographic and clinical characteristics of adults with antineutrophil cytoplasmic antibody-associated vasculitis

		White (N=669)	Black or African American (N=65)
Gender			
Female		377 (56.4%)	43 (66.2%)
Male		292 (43.6%)	22 (33.8%)
Ethnicity			
Non-Hispanic/Latino		587 (87.7%)	62 (95.4%)
Hispanic/Latino		54 (8.1%)	3 (4.6%)
Unknown		28 (4.2%)	0 (0.0%)
Age			
Age at diagnosis, years (mean ± SD)		64 ± 16	55 ± 15
Patient diagnosis and condition			
Dependence on renal dialysis (Z99.2)		89 (13.3%)	14 (21.5%)
Diabetes		270 (40.4%)	29 (44.6%)
Crescentic glomerulonephritis (N05.7)		38 (5.7%)	3 (4.6%)
Anti-glomerular basement membrane positivity/Goodpasture syndrome (M31.0)		28 (4.2%)	2 (3.1%)
Clinical laboratory tests and biomarkers			
Tested myeloperoxidase (MPO) (Logical Observation Identifiers Names and Codes (LOINC) 17316-1)		19 (2.8%)	2 (3.1%)
Abnormal MPO (LOINC 17316-1)		5 (0.7%)	0 (0.0%)
Tested proteinase 3 (PR3) (LOINC 6968-2, 63310-7, 29644-2)		142 (21.2%)	13 (20.0%)
Abnormal PR3 (LOINC 6968-2, 63310-7, 29644-2)		38 (5.7%)	5 (7.7%)
Abnormal creatinine - blood (LOINC 2160-0)		422 (63.1%)	35 (53.8%)
Abnormal urine protein-to-creatinine ratio or urine albumin-to-creatinine ratio		121 (18.1%)	13 (20.0%)
Clinical signs and symptoms			
Proteinuria (R80.9)		131 (20.0%)	13 (20.0%)
Hematuria (R31)		188 (28.1%)	25 (38.5%)
Values are n (%) unless otherwise indicated. Percentages are calculated within race categories. Antineutrophil cytoplasmic antibody serologies were incompletely captured in the electronic health record and are presented descriptively.			

Mean age at diagnosis differed by race, with White patients diagnosed at a mean age of 64 ± 16 years and Black patients at 55 ± 15 years. Renal involvement at presentation was common across the cohort. Dialysis dependence was documented in 13.3% of White patients and 21.5% of Black patients. Abnormal serum creatinine was present in 63.1% of White patients and 53.8% of Black patients.

Proteinuria was present in 20% of both White and Black patients, whereas hematuria was observed in 28.1% of White patients and 38.5% of Black patients. Comorbid diabetes mellitus was documented in 40.4% of White patients and 44.6% of Black patients.

ANCA serologic testing was incompletely captured within the EHR. Testing for PR3 antibodies was documented in 142 (21.2%) White patients and 13 (20.0%) Black patients, with abnormal results recorded in 38 (5.7%) and 5 (7.7%), respectively. MPO testing was documented in fewer than 3% of patients in either group.

Comparative analyses of age at diagnosis and renal outcomes by race are summarized in Table 2. Mean age at diagnosis was lower among Black patients than White patients (55 ± 15 vs 64 ± 16 years; p<0.001). Differences in renal outcomes were observed between groups. Dialysis dependence was documented in 21.5% of Black patients and 13.3% of White patients, corresponding to an OR of 1.79 (95% CI: 0.88-3.45; p=0.090). Hematuria was documented in 38.5% of Black patients compared with 28.1% of White patients, with an OR of 1.59 (95% CI: 0.90-2.78; p=0.086).

**Table 2:** Racial differences in key clinical outcomes at presentation

	White (N=669)	Black (N=65)	Odds ratio	95% confidence interval	p-value
Age at diagnosis, years	64 ± 16	55 ± 15	NA	NA	<0.001
Dialysis dependence	13.3%	21.5%	1.79	(0.88-3.45)	0.090
Hematuria	28.1%	38.5%	1.59	(0.90-2.78)	0.086
Odds ratios are presented as Black vs White. Age comparison was performed using a Welch t-test based on summary statistics, and categorical comparisons were performed using Fisher’s exact test.					

## Discussion

Among adults with antineutrophil cytoplasmic antibody-associated vasculitis (AAV) captured in an EHR cohort, we observed clinically meaningful differences in age at diagnosis and renal disease severity between Black and White patients. Black patients were diagnosed at a younger mean age and exhibited higher frequencies of dialysis dependence and hematuria at presentation. These findings suggest that heterogeneity in disease presentation within the spectrum of AAV highlights the importance of examining severity at presentation alongside demographic characteristics.

Age at diagnosis and life-course disease burden

The nearly decade-younger mean age at diagnosis among Black patients falls within the expected epidemiologic range for AAV onset but may have important implications for cumulative disease burden over time [1-3]. Prior studies indicate that AAV most commonly presents in mid-to-late adulthood and that a younger age at onset does not necessarily reflect milder disease or more favorable outcomes [2,4]. Earlier diagnosis may instead be associated with prolonged exposure to disease activity, immunosuppressive therapy, and treatment-related toxicity, all of which contribute to long-term morbidity [8,10].

Renal disease severity at presentation

Markers of renal involvement at presentation differed across racial groups and provided important context for understanding disease burden. Dialysis dependence and hematuria were more frequently observed among Black patients though not statistically significant in this cohort. These findings are consistent with prior literature demonstrating that dialysis dependence and active urinary sediment at diagnosis reflect severe renal involvement and are strongly associated with adverse kidney outcomes, independent of subsequent treatment strategy [7,8,10].

Renal severity in the context of contemporary AAV management

Despite advances in AAV treatment, including rituximab-based induction strategies and glucocorticoid-sparing approaches, the severity of renal disease at presentation remains a major determinant of prognosis [8,12,15]. Current Kidney Disease: Improving Global Outcomes (KDIGO) guidelines recognize that kidney involvement in AAV varies widely and that dialysis dependence has important implications for prognosis and management [12,15,16]. Differences in renal severity at presentation and histopathologic findings have downstream implications for treatment intensity, duration of immunosuppression, and likelihood of renal recovery.

Disease heterogeneity and limitations of serologic data

Increasing evidence supports the prognostic relevance of ANCA serotype, particularly distinctions between PR3-ANCA and MPO-ANCA disease, which may be more informative than clinical phenotype alone in predicting relapse risk and long-term outcomes [4,7,13]. In this cohort, ANCA serologic testing was incompletely captured, limiting the ability to examine serotype-specific patterns and complete validation of diagnoses. Unfortunately, in Epic Cosmos, we were unable to classify the phenotypic distribution of patients with granulomatosis with polyangiitis (GPA), microscopic polyangiitis (MPA), and eosinophilic GPA (EGPA) within the study cohort. This limitation reflects real-world diagnostic workflows in large EHR datasets and highlights the challenge of fully characterizing disease biology in retrospective, population-based studies, further emphasizing the importance of real-world datasets. The authors are currently exploring these questions within our own health system, and this exploratory cohort was designed to explore hypotheses and lead to further investigation. 

Representation and epidemiologic considerations

Although Black patients represented a small proportion of the cohort, they had a younger age and were more likely to require dialysis and have hematuria, although these results were not statistically significant. Prior epidemiologic studies have consistently reported lower observed prevalence of AAV among Black populations, with most data derived from cohorts of European ancestry [1,3,4]. The present findings contribute to a limited but growing literature suggesting that underrepresentation in rare disease cohorts does not equate to lower disease burden and may coexist with markers of more severe presentation [9,14].

Implications for research and care delivery

Guidelines emphasize early recognition, prompt initiation of immunosuppressive therapy, and multidisciplinary management as central to improving outcomes in AAV [2,12,15]. The renal-predominant and severe presentations observed in this cohort reinforce the importance of early kidney evaluation and vigilant clinical suspicion [5,9].

Limitations

This study is limited by its retrospective design and reliance on EHR-derived data, which may be subject to coding inaccuracies and incomplete capture of clinical variables, including accurate information about treatments and the phenotypic presentation of their vasculitis. Specifically, despite a higher percentage of dialysis dependence noted in African American patients, they were less likely to have been coded as having an abnormal creatinine. We were unable to classify the phenotypic distribution of patients with GPA, MPA, and EGPA within the study cohort, which may have skewed our results, as patients with EGPA are less likely to have renal involvement. Due to the nature of the EHR data, we were not able to validate AAV diagnoses beyond ICD-10 codes, increasing the risk of misclassification bias. Furthermore, dialysis dependence was more commonly described than documented crescentic glomerulonephritis, underscoring the difficulty in capturing biopsy findings in the EHR and limiting more analysis of renal severity. Also, this study was limited as it only captured patients whose care occurred in a system using the Epic EHR in the United States, which limits generalizability

## Conclusions

Differences in age at diagnosis and renal disease severity were observed between Black and White adults with ANCA-associated vasculitis. These findings highlight that underrepresentation in rare disease cohorts may coexist with markers of substantial disease burden at presentation. Future studies incorporating longitudinal outcomes, biopsy data, and more complete serologic characterization are needed to clarify mechanisms underlying these differences and to inform earlier, risk-informed, and equitable approaches to AAV care.
